# Effects of a local single dose administration of growth hormone on the osseointegration of titanium implants

**DOI:** 10.4317/medoral.25164

**Published:** 2022-02-20

**Authors:** João Ricardo Almeida Grossi, Marcelo Parra, Ernesto B Benalcázar-Jalkh, Allan Fernando Giovanini, João César Zielak, Aline Monise Sebstiani, Carla Castiglia Gonzaga, Paulo G Coelho, Lukasz Witek, Tatiana Miranda Deliberador

**Affiliations:** 1 School of Health Science, Positivo University, Curitiba, Paraná, Brazil; 2 Department of Biomaterials, New York University College of Dentistry, New York, USA; 3 PhD Program in Morphological Sciences, Center of Excellence in Morphological and Surgical Studies Faculty of Medicine, Universidad de La Frontera, Temuco, Chile; 4 University of Sao Paulo - Bauru School of Dentistry, Department of Prosthodontics and Periodontology, Bauru, Brazil; 5 Department of Mechanical and Aerospace Engineering, New York University Tandon School of Engineering, Brooklyn, USA; 6 Hansjörg Wyss Department of Plastic Surgery, NYU Langone Health, New York; 7 Department of Biomedical Engineering, New York University Tandon School of Engineering, Brooklyn, USA

## Abstract

**Background:**

The aim of the present study was to evaluate the effect of different concentrations of growth hormone (GH) on endosteal implant’s surface at the early stages of osseointegration.

**Material and Methods:**

Sixty tapered acid-etched titanium implants were divided into four groups: i) Collagen, used as a control group; and three experimental groups, where after collagen coating, GH was administered directly to the surface in varying concentrations: ii) 0.265 mg, iii) 0.53 mg, and iv) 1 mg. Implants were placed in an interpolated fashion in the anterior flange of C3, C4 or C5 of 15 sheep with minimum distance of 6 mm between implants. After 3-, 6- and 12-weeks of healing samples were harvested, histologically processed, qualitatively and quantitatively assessed for bone-to-implant contact (BIC) and bone area fraction occupancy (BAFO).

**Results:**

Statistical analysis as a function of time *in vivo* and coating resulted in no significant differences for BIC and BAFO at any evaluation time point. Histological evaluation demonstrated similar osseointegration features for all groups with woven bone formation at 3 weeks and progressive replacement of woven for lamellar bone in close contact with the implant surface and within the implant’s threads.

**Conclusions:**

A single local application of growth hormone to the surface of titanium implants did not yield improved implant osseointegration independent of healing time.

** Key words:**Growth hormone, osseointegration, low density bones, metallic implants, sheep, bone-to-implant contact.

## Introduction

Osteointegration of endosteal implants is dependent on several conditions. A variety of factors have been evaluated in an effort to improve/accelerate osseointegration while attempting to reduce the time required for implant loading (1). Implant design, instrumentation protocols and surface treatments have been the primary focus of dental implant research, with particular attention given to modifications of the implant’s surface in order to stimulate cell migration and differentiation at the bone-to-implant interface (2). Although significant improvements have been achieved to reduce healing times of implant therapy, poor quality bone (e.g., low density) and systemic conditions (i.e., diabetes, metabolic syndrome, use of tobacco) are still considered challenging scenarios for implant dentistry, demanding extended healing periods and being frequently associated with higher risk for early implant failure (3,4).

Among the modalities proposed to overcome bone healing impairment commonly associated with low quality bone, related with implant failures has been the application of natural substances directly to the implant's surface to stimulate the biological interaction at the osteotomy site. Such stimulation may result in increased osseoconductibility, enhanced mineral deposition, and subsequently, expediated, predicTable, long-lasting osseointegration (5-7). Growth hormone (GH), a 70 amino acid polypeptide chain originating from the pituitary gland, has demonstrated to have a significant impact in both osteoblast and osteoclast activity (8,9). Furthermore, it has been suggested to positively influence new bone formation and increase the cortical bone mass during bone regeneration procedures (10,11). Additionally, GH has been demonstrated to improve calcium absorption and vitamin D levels (12). In previous *in vivo* studies with small animal models, the systemic administration of GH significantly increased different osseointegration parameters of endosteal implants in osteoporotic subjects (8,13-17). GH has also been suggested to have a local effect on bone remodeling through the stimulation of the synthesis of collagen, osteocalcin and alkaline phosphatase. This mechanism was used in previous research to enhance the substitution of biomaterial by bone through speeding up the remodeling process (18). Additionally, when administered locally at the implant site, GH has demonstrated to increase hard tissue mineralization (14,19-21). However, contradictory results, such as lack of osteogenic stimulus (22), and adverse effects in bone healing with the application of GH dependent on onset and duration of administration have been previously reported in literature (23).

While different compounds have demonstrated a positive effect in stimulating new bone formation and mineralization around implanted devices (24), there remains a paucity in the literature regarding the effects of local administration of GH at implant placement. The current study aimed to assess the effect of different concentrations applied as a local single dose on the osseointegration parameters of titanium implants in low density bone at 3-, 6- and 12-weeks.

## Material and Methods

The study was conducted after receiving the approval of the Research Ethics Committee on Animal Use (CEUA) at the Positivo University (Protocol 274/2015) in accordance with the provisions of the Arouca Law (11794/2008) and designed according to ARRIVE guidelines.

- Study design

Fifteen adult female sheep (~24 months), with an average weight of 65 kg were utilized in the present study. The sheep’s cervical spine was the chosen model due to its low-density bone conFiguration and size, which permitted all experimental groups to be nested within each subject. Prior to surgery, general anesthesia was induced with Sodium Pentothal (15-20mg/kg) in Normasol solution into the jugular vein and maintained with isoflurane (1.5-3%) in O2/N2O (50/50), with continuous monitoring of the vital functions. After the anesthesia, the surgical site was shaved, and disinfected with betadine. A ~15 cm incision was made along the midline, starting 5 cm below the cricoid cartilage. Once the incision was performed, blunt dissection was used to gain access to the vertebrae's anterior flange.

Sixty tapered acid-etched dental implants (Novo Colosso Emfils Colosso, Itu, São Paulo, Brazil ; 4mm diameter and 10 mm length) were divided in four groups: i) Collagen, used as the study control group (Collagen Type I Corning Inc., Corning, NY, USA); and three experimental groups, where after collagen coating, GH was applied directly to the implant’s surface of different concentrations: ii) 0.265 mg iii) 0.53 mg iv) and 1 mg. Implants were placed in an interpolated fashion in the anterior flange of C3, C4 or C5 into osteotomies prepared using conventional surgical drilling in a 3-step series twist drills, as recommended by the manufacturer (Emfils Colosso Drills, Itu, Brazil). All the implants were placed with a minimum distance of 6 mm between each other.

A simple suture of the muscle’s fascia was performed with 2.0 polyglactin absorbable suture (Vicryl Ethicon, São Paulo, SP, Brazil), followed by a continuous skin suture with 2.0 nylon thread (Shalon Surgical Threads Ltda, São Luiz de Montes Belos, GO, Brazil). After surgery, ketoprofen 10 % (Biofen, Biofarm Química e Farmacêutica LTDA, Jaboticabal, SP, Brazil) was administered in a dose of 3 mg/kg intramuscularly once a day for 3 days and enrofloxacin 10 % (injecTable Chemitril 10 %, Chemitec Agro Veterinária LTDA, São Paulo, SP, Brazil) in a dose of 2.5 mg/kg once a day for 5 days intramuscularly. All the animals were offered water and food *ad libitum* and monitored daily for pain, lameness, open wounds or any other signs of complications.

According to protocol the animals were euthanized 3-, 6- and 12-weeks after surgery. Euthanasia was performed by overdose of sodium thiopental (Thiopentax, Cristália, São Paulo, SP, Brazil) and the implants as well as the surrounding bone tissue was removed *en bloc*k.

- Sample preparation and histologic and histomorphometric analysis

En block samples were gradually dehydrated in a series of alcohol solutions ranging from 70-100% ethanol and then embedded in a methyl methacrylate-based resin. Embedded blocks were then cut into thin sections (~300μm) using a diamond saw aiming at the implant center (Isomet 2000, Buehler Ltd., IL, USA). The sections were ground using a grinding machine (Metaserv 3000, Buehler, Lake Bluff, IL, USA) under continuous water irrigation with a series of SiC abrasive paper until achieving a thickness of approximately 100 μm thick. The sections were stained with Stevenel's Blue and Van Giesons Picro Fuschin, and scanned using an automated microscope system (Aperio Technologies, Vista, CA).

Digital micrographs were subjected to qualitative observations to compare histological features and osseointegration patterns among groups. Histomorphometric quantitative analysis was performed in means of Bone-to-implant contact (BIC), measured as the percentage of bone in direct contact over the entire implant surface, and bone area fraction occupancy (BAFO), measured as the percentage of bone growth within the implant threads. All evaluations were performed by a calibrated, single blind examiner using a specific computer software (ImageJ, NIH, Bethesda, MD).

- Statistical analysis

Histomorphometric analyses data are presented as mean values with corresponding 95% confidence interval values (mean ± CI). The %BIC and % BAFO data were analyzed using a linear mixed model with fixed factors of time *in vivo* (3-, 6- and 12-weeks) and coating (Collagen, 0.265 mg, 0.53 mg and 1 mg). All analyses were completed with IBM SPSS (v23, IBM Corp., Armonk, NY).

## Results

No signs of complications, infection or disease were observed at any follow up period.

- Histomorphometric analyses

**Figure 1:**
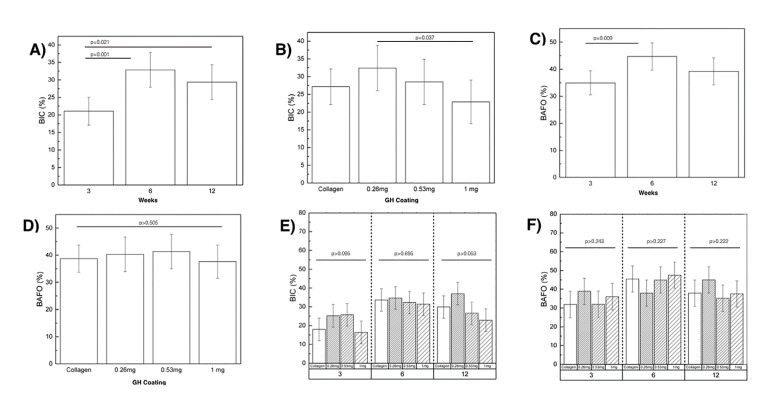
Statistical analyses of Bone to Implant Contact and Bone area fraction occupancy statistically analyzed collapsed over time (A and C), and over coating (B and D). (E) Bone to Implant Contact (%) and (F) Bone area fraction occupancy (%) of all experimental groups at each time point evaluation. Error bars represent 95% confidence interval (CI).

Statistical evaluation of BIC as a function of time with data collapsed over coating demonstrated significant differences (Fig. 1). BIC values were significantly lower at three weeks (21.07% ± 4.63) compared to six weeks (32.85% ± 5.21) and twelve weeks *in vivo* (29.35% ± 4.96) (*p*=0.021) (Fig. 1). While evaluation of BIC as a function of coating collapsed over time did not depict statistical differences between experimental and control group (*p*=0.106), the 0.265 mg group (32.42% ± 5.91) did yield significantly higher BIC values in comparison to implants treated with 1 mg of GH (22.87%±5.55; *p*=0.037) (Fig. 1).

Analysis of BAFO as a function of time *in vivo*, followed similar trends as with BIC, when data was collapsed over coating, significantly lower BAFO values were observed at 3-weeks (34.95% ± 4.89) compared to values at 6-weeks (44.69% ± 5.51; *p*=0.013), with no significant differences at 12-weeks (40.68% ± 5.25; *p*=0.246) (Fig. 1). Evaluation of BAFO as a function of coating, collapsed over time, did not yield statistical differences (*p*=0.505), with an average BAFO value of 39.94% ± 6.01 (Fig. 1).

When considering a two-level analysis of time *in vivo* and coating, no significant differences were observed for BIC (Fig. 1) and BAFO (Fig. 1) at any time point. While no statistical differences were detected among groups, significance reached marginal values at 12 weeks in the comparison between 0.265 mg and 1 mg groups (*p*=0.053).

- Histological analysis

**Figure 2:**
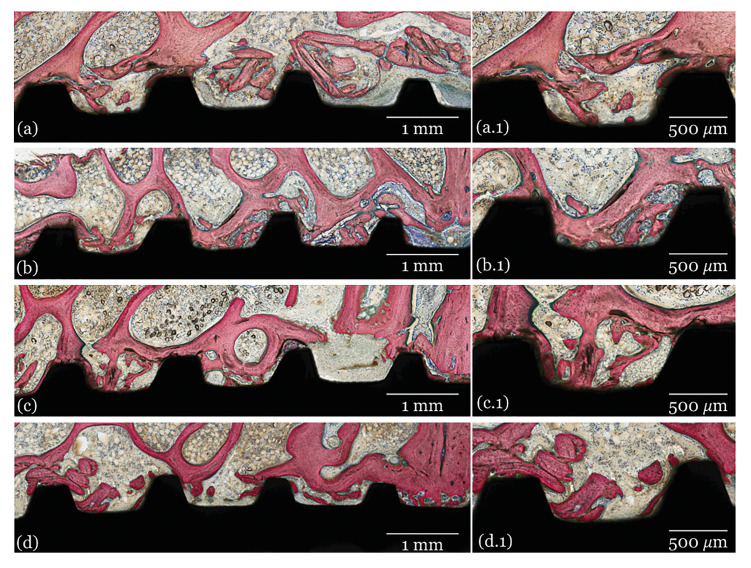
Representative optical micrographs of healing chambers (1) and bone/implant interface at higher magnification (2) of implant groups at 3 weeks: A) Collagen, B) 0.265 mg GH C) 0.53 mg GH and D) 1 mg GH.

**Figure 3:**
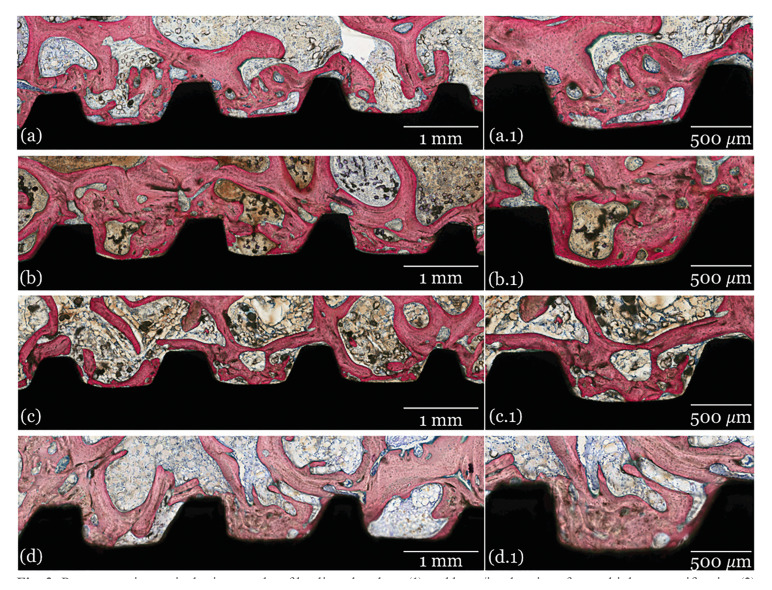
Representative optical micrographs of healing chambers (1) and bone/implant interface at higher magnification (2) of implant groups at 6 weeks: A) Collagen, B) 0.265 mg GH C) 0.53 mg GH and D) 1 mg GH.

**Figure 4:**
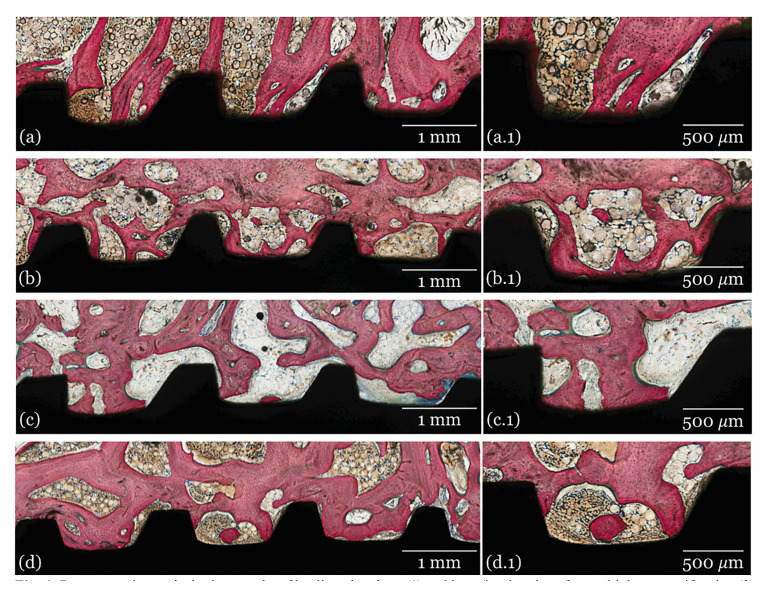
Representative optical micrographs of healing chambers (1) and bone/implant interface at higher magnification (2) of implant groups at 12 weeks: A) Collagen, B) 0.265 mg GH C) 0.53 mg GH and D) 1 mg GH.

The histological images of all groups at 3-, 6- and 12- weeks are presented in Fig. 2, Fig. 3 and Fig. 4, respectively. Qualitative evaluation of the samples supported the findings of the histomorphometrical analyses. All groups presented similar osseointegration features in trabecular bone, where an intramembranous-type healing pattern was observed. At 3 weeks, the histological evaluation depicted similar osseointegration features for all groups, with similar woven bone formation in close contact with the implant surface and within the implant’s threads. At 6- and 12-weeks, progressive replacement of woven bone with lamellar bone was observed with evidence of similar tissue maturation and mineralization independent of the presence/absence or doses of GH coating.

## Discussion

The initial bone healing process immediately after surgical trauma is critical to maintain the initial implant stability within the osteotomy (25). Following surgical instrumentation, the early catabolic events during bone regeneration result in a decreasing mineral density in the immediate vicinity. Therefore, the local administration of natural substances that stimulate mineral deposition have been proposed to improve bone formation and its subsequent maturation in the early stages of osseointegration. In the present study, the application of different concentrations of GH directly to the implant surface was ineffective to promote significant differences in the evaluated osseointegration parameters. The absence of quantitative and qualitative differences between the control and the experimental groups led us to accept the null hypothesis of the study.

BIC and BAFO have long been established quantitative parameters in scientific literature in an effort to evaluate the levels/degrees of osseointegration of implanted devices (26,27). BAFO reflects the bone occupancy fraction, which can be occupied by newly formed bone via distance osteogenesis or contact osteogenesis, such as for bone fragments compressed between bone wall. BIC represents new bone formation in direct contact with the implant surface, which has been related to contact osteogenesis. In the present study the primary factor that influenced BIC and BAFO was the time *in vivo*, where a significant increment of both parameters was observed over time. Woven bone formation and its progressive mineralization, organization and replacement by lamellar bone is expected to occur at the implant surface and within the implant’s healing chambers. In low density, quality bone, this process has been suggested to demand longer healing times and to be a risk factor for early implant failure.

Previous literature has suggested that the local application of 4UI (1.2 mg) of lyophilized GH powder at the time of implant placement could enhance peri-implant bone reaction (14). This mechanism has been associated with the stimulation of local calcium availability to increase the mineralization of the newly formed osteoid tissue (20). Additionally, it has been suggested that local GH administration may stimulate osteocytes osteolysis and thus increase calcium perilacunar availability (14). A handful of *in vivo* studies in small rodents have reported a positive effect of the local administration of GH to stimulate bone formation in critical defects and around implanted devices (20,21,28). However, controversies have been reported in the effect of local GH to promote repair in bone defects (22,29). In an *in vivo* study performed in dogs, Theyse *et al*. studied the effects of systemic and local administration of GH for the treatment of critical-sized bone defects. The results of the study suggested that systemic administration of GH enhanced the healing of bone defects with no osteogenic effect of the local administration of GH to the defect site (29).

When applied to the osteotomy site prior to implant placement in osteoporotic bones of New Zealand rabbits, local GH has been suggested to increase bone to implant contact relative to untreated sites (28). While some literature has reported the effects of GH in small animal models, the benefits associated to local administration of GH at implant surface remains unclear (22,30). While the results of the present study did not evidence advantages in the use of GH, further studies are warranted where its clinical application in osteoporotic or compromised subjects (e.g., diabetes, hormonal alterations) can be considered, as well as including different GH concentrations.

## Conclusions

Single local application of GH on the surface of titanium implants did not facilitate or improve osseointegration at any time point evaluation.

## References

[1] Bonfante EA, Jimbo R, Witek L, Tovar N, Neiva R, Torroni A (2019). Biomaterial and biomechanical considerations to prevent risks in implant therapy. Periodontol 2000.

[2] Coelho PG, Jimbo R, Tovar N, Bonfante EA (2015). Osseointegration: hierarchical designing encompassing the macrometer, micrometer, and nanometer length scales. Dent Mater.

[3] Goiato MC, dos Santos DM, Santiago JF Jr, Moreno A, Pellizzer EP (2014). Longevity of dental implants in type IV bone: a systematic review. Int J Oral Maxillofac Surg.

[4] Staedt H, Rossa M, Lehmann KM, Al-Nawas B, Kammerer PW, Heimes D (2020). Potential risk factors for early and late dental implant failure: a retrospective clinical study on 9080 implants. Int J Implant Dent.

[5] Marin C, Jimbo R, Lorenzoni FC, Witek L, Teixeira H, Bonfante E (2013). Bone-Forming Capabilities of a Newly Developed NanoHA Composite Alloplast Infused with Collagen: A Pilot Study in the Sheep Mandible. Int J Dent.

[6] Osagie-Clouard L, Sanghani A, Coathup M, Briggs T, Bostrom M, Blunn G (2017). Parathyroid hormone 1-34 and skeletal anabolic action: The use of parathyroid hormone in bone formation. Bone Joint Res.

[7] Yoo D, Tovar N, Jimbo R, Marin C, Anchieta RB, Machado LS (2014). Increased osseointegration effect of bone morphogenetic protein 2 on dental implants: an in vivo study. J Biomed Mater Res A.

[8] Klein IE (1975). The effect of thyrocalcitonin and growth hormones on bone metabolism. J Prosthet Dent.

[9] Ohlsson C, Vidal O (1998). Effects of growth hormone and insulin-like growth factors on human osteoblasts. Eur J Clin Invest.

[10] Bak B, Andreassen TT (1991). The effect of growth hormone on fracture healing in old rats. Bone.

[11] Kim Y, Hong JW, Chung YS, Kim SW, Cho YW, Kim JH (2014). Efficacy and safety of sustained-release recombinant human growth hormone in Korean adults with growth hormone deficiency. Yonsei Med J.

[12] Varkey M, Gittens SA, Uludag H (2004). Growth factor delivery for bone tissue repair: an update. Expert Opin Drug Deliv.

[13] Ohlsson C, Bengtsson BA, Isaksson OG, Andreassen TT, Slootweg MC (1998). Growth hormone and bone. Endocr Rev.

[14] Tresguerres IF, Blanco L, Clemente C, Tresguerres JA (2003). Effects of local administration of growth hormone in peri-implant bone: an experimental study with implants in rabbit tibiae. Int J Oral Maxillofac Implants.

[15] Nielsen HM, Bak B, Jorgensen PH, Andreassen TT (1991). Growth hormone promotes healing of tibial fractures in the rat. Acta Orthop Scand.

[16] Gomez-Moreno G, Cutando A, Arana C, Worf CV, Guardia J, Munoz F (2009). The effects of growth hormone on the initial bone formation around implants. Int J Oral Maxillofac Implants.

[17] Stenport VF, Olsson B, Morberg P, Tornell J, Johansson CB (2001). Systemically administered human growth hormone improves initial implant stability: an experimental study in the rabbit. Clin Implant Dent Relat Res.

[18] Guicheux J, Gauthier O, Aguado E, Pilet P, Couillaud S, Jegou D (1998). Human growth hormone locally released in bone sites by calcium-phosphate biomaterial stimulates ceramic bone substitution without systemic effects: a rabbit study. J Bone Miner Res.

[19] Munoz F, Lopez-Pena M, Mino N, Gomez-Moreno G, Guardia J, Cutando A (2012). Topical application of melatonin and growth hormone accelerates bone healing around dental implants in dogs. Clin Implant Dent Relat Res.

[20] Tresguerres IF, Alobera MA, Baca R, Tresguerres JA (2005). Histologic, morphometric, and densitometric study of peri-implant bone in rabbits with local administration of growth hormone. Int J Oral Maxillofac Implants.

[21] Tresguerres IF, Clemente C, Donado M, Gomez-Pellico L, Blanco L, Alobera MA (2002). Local administration of growth hormone enhances periimplant bone reaction in an osteoporotic rabbit model. Clin Oral Implants Res.

[22] Calvo-Guirado JL, Mate-Sanchez J, Delgado-Ruiz R, Ramirez-Fernandez MP, Cutando-Soriano A, Pena M (2011). Effects of growth hormone on initial bone formation around dental implants: a dog study. Clin Oral Implants Res.

[23] Bak B, Jorgensen PH, Andreassen TT (1991). The stimulating effect of growth hormone on fracture healing is dependent on onset and duration of administration. Clin Orthop Relat Res.

[24] Alenezi A, Chrcanovic B, Wennerberg A (2018). Effects of Local Drug and Chemical Compound Delivery on Bone Regeneration Around Dental Implants in Animal Models: A Systematic Review and Meta-Analysis. Int J Oral Maxillofac Implants.

[25] Gabet Y, Kohavi D, Voide R, Mueller TL, Muller R, Bab I (2010). Endosseous implant anchorage is critically dependent on mechanostructural determinants of peri-implant bone trabeculae. J Bone Miner Res.

[26] Beutel BG, Danna NR, Granato R, Bonfante EA, Marin C, Tovar N (2016). Implant design and its effects on osseointegration over time within cortical and trabecular bone. J Biomed Mater Res B Appl Biomater.

[27] Wennerberg A, Albrektsson T (2009). Effects of titanium surface topography on bone integration: a systematic review. Clin Oral Implants Res.

[28] Martin-Monge E, Tresguerres IF, Clemente C, Tresguerres JA (2017). Local Application of Growth Hormone to Enhance Osseointegration in Osteoporotic Bones: A Morphometric and Densitometric Study. Int J Oral Maxillofac Implants.

[29] Theyse LF, Oosterlaken-Dijksterhuis MA, van Doorn J, Dhert WJ, Hazewinkel HA (2006). Growth hormone stimulates bone healing in a critical-sized bone defect model. Clin Orthop Relat Res.

[30] Eriksen EF, Kassem M, Langdahl B (1996). Growth hormone, insulin-like growth factors and bone remodelling. Eur J Clin Invest.

